# A general recipe to observe non-Abelian gauge field in metamaterials

**DOI:** 10.1515/nanoph-2024-0414

**Published:** 2024-10-30

**Authors:** Bingbing Liu, Tao Xu, Zhi Hong Hang

**Affiliations:** School of Physical Science and Technology and Collaborative Innovation Center of Suzhou Nano Science and Technology, 12582 Soochow University, Suzhou 215006, China; AVIC Leihua Electronic Technology Research Institute, Wuxi 214082, China; Institute for Advanced Study and Jiangsu Key Laboratory of Frontier Material Physics and Devices, 12582 Soochow University, Suzhou 215006, China; Aviation Key Laboratory of Science and Technology on AISSS, Wuxi 214082, China

**Keywords:** metamaterials, non-Abelian gauge field, *Zitterbewegung*

## Abstract

Recent research on non-Abelian phenomena has cast a new perspective on controlling light. In this work, we provide a simple and general approach to induce non-Abelian gauge field to tremble the light beam trajectory. With in-plane duality symmetry relaxed, our theoretical analysis finds that non-Abelian electric field can be synthesized through a simple real-space rotation of any biaxial material. With orthogonal optical modes excited, their interference leads to an oscillation of the propagating optical beam, which is a direct consequence of the emergence of non-Abelian electric field, influencing light in a manner similar with how electric fields act on charged particles. Our microwave experiments provide unambiguous evidence to the observation of such an optical *Zitterbewegung* effect where excellent agreement can be found between theorical derivation, numerical simulations and experiments. By extending the idea to optical regime using natural material, we here provide another example to shake the general intuition that light travels in straight lines in homogeneous media.

## 1 Introduction

The concept of gauge potential originates from electromagnetism [1], [2], and was further generalized to non-Abelian groups by Yang and Mills [3]. Because of its non-commutativity feature, non-Abelian phenomena are universal in physics, ranging from rigid body rotation to cold atoms, from particle physics to topology physics [4], [5], [6]. For instance, Berry connection in topology physics is actually the scalar form of matrix-valued gauge potentials, and its associated Berry curvature can be understood as the magnetic field in momentum space. Thus, topology physics can be regarded as a consequence of Abelian or non-Abelian gauge fields in momentum space. The braiding property arising from the non-commutativity of non-Abelian topological charges has important implications for quantum computing and communications [7] as well. On the other hand, topological photonics [8], has become a hot research field because of its perfect performance in demonstrating fascinating theories in topology physics. Especially, the non-Abelian counterpart of Berry curvature, has successfully led to novel physical effects in light and sound [9], [10], [11], [12], [13], [14], [15].

Other than works in momentum space, the convenience of sample preparation in optics makes the manipulation of non-Abelian gauge field in real space extremely simple while different recipes to observe non-Abelian phenomena are proposed using microcavities [16], metamaterials [17], fiber optics [18], electric circuits [19], and two-dimensional (2D) materials [20], making optics an ideal platform to visualize for instance the Aharonov–Bohm effect and *Zitterbewegung* (ZB) of light beams. Synthetic non-Abelian gauge fields in real space not only offer a straightforward way to manipulate light but also possess great physical significance, as they directly influence light in a manner similar to how electromagnetic fields act on charged particles.

Birefringent crystals are a common type of optical materials and can play an important role in the manipulation of electromagnetic waves, where intriguing phenomena such as negative refraction [21], ghost waves [22], Dyakonov surface waves [23], and macroscopic cloaking [24], [25] have been demonstrated using natural birefringent crystals. However, the minute difference in refractive indices in natural uniaxial or biaxial crystals limits their broad applications which can be easily compensated by the introduction of metamaterials. Providing unprecedently customization of effective permittivity and permeability, metamaterials contribute enormously to light manipulations, including negative refraction [26], invisibility cloaking [27], electromagnetic absorption [28] and topologically protected interface states [29], [30], [31]. Abelian [32], [33] and non-Abelian gauge fields [17] have also been proposed to realize using metamaterials. However, the in-plane duality symmetry [34] to induce gauge field demands a tailored 18 parameters in the anisotropic permittivity and permeability tensors which is extremely challenging in metamaterial design. Even if parameters with no magnetic response are chosen, duality symmetry will impose additional constraints on the material parameters, which makes the observation of the interesting non-Abelian gauge field related properties rarely reported not to mention potential applications.

**Figure 1:**
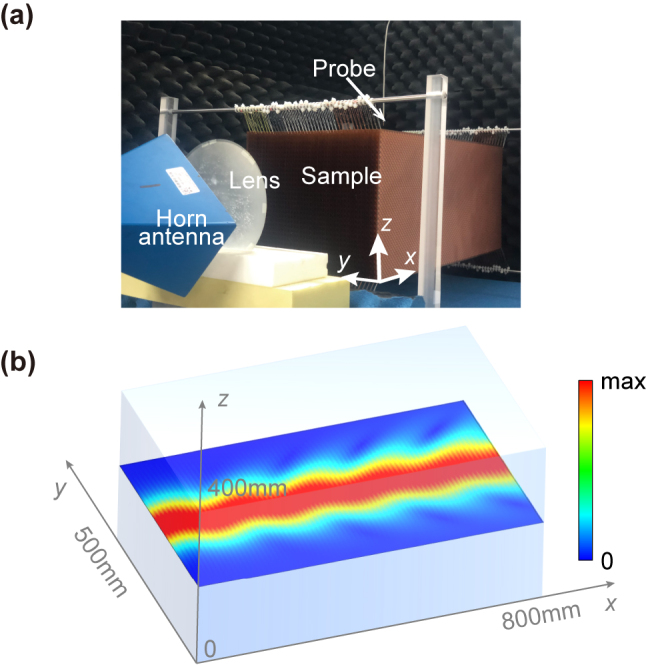
Observation of ZB effect in biaxial medium. (a) Experimental setup to visualize ZB effect in metamaterials. (b) Simulated results showing that the non-Abelian gauge field induce an oscillation of microwave incident beam at 12.900 GHz, using the exact experimental parameters.

In this work, we present a general recipe to observe non-Abelian electric field. By rigorous derivations of the light propagation inside an arbitrary biaxial dielectric medium, we verify that a simple rotation with any chosen angle in real space can effectively generate non-Abelian electric fields to alter the electromagnetic wave propagation and induce a ZB motion of the incident beam. In our microwave experiment (Figure 1), with certain polarization of electromagnetic wave, the incident beam was measured to oscillate along the direction perpendicular to its propagation. Good consistence can be found between our theoretical analysis, numerical simulations and experimental observations, which make this work, to the best of our knowledge, the first experimental effort to visualize non-Abelian electric field. Moreover, this idea can be extended to natural material, and make it another example to break the notion that light spreads along a straight line inside homogeneous media other than accelerating waves [35].

## 2 Theoretical analysis

**Figure 2:**
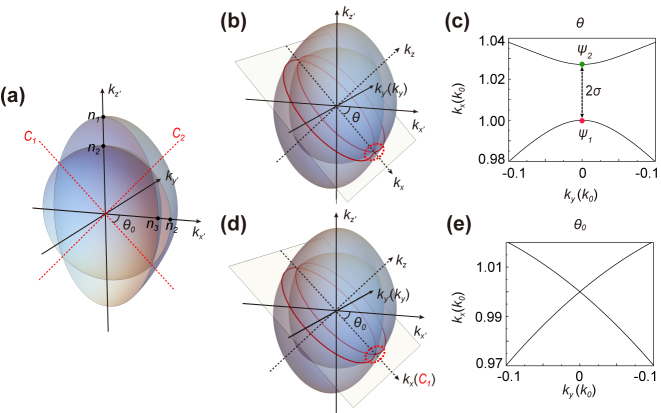
Dispersion relations of biaxial crystal. (a) EFSs (cross-section view from inside) of biaxial crystal in the principle axis system with $n_{1}=\sqrt{\varepsilon_{1}}$ , $n_{2}=\sqrt{\varepsilon_{2}}$ and $n_{3}=\sqrt{\varepsilon_{3}}$ being the three principle refractive indices, and the red dashed lines correspond to the two optical axes *C*_1_ and *C*_2_; (b) EFCs (red lines) in the new *k*_*x*_–*k*_*y*_ plane obtained by a coordinate rotation of angle *θ* along *k*_*y*′_ axis; (c) the enlarged view of (b) near *k*_*y*_ = 0 (regions marked by the dashed red ellipse); (d) EFCs in *k*_*x*_ *–* *k*_*y*_ plane at a rotation angle *θ* = *θ*_0_; (e) enlarged view of (d) near *k*_*y*_ = 0.

We start by considering a general biaxial crystal with a relative permittivity tensor $\left(\begin{array}{ccc} \varepsilon_{1} & 0 & 0\\ 0 & \varepsilon_{2} & 0\\ 0 & 0 & \varepsilon_{3} \end{array}\right)$ in the principle coordinate system *x*′–*y*′–*z*′ while unity relative permeability is maintained. Without losing generality, we use *ɛ*_1_ > *ɛ*_2_ > *ɛ*_3_. As can be seen in Figure 2(a), the equifrequency surfaces (EFSs) of such a biaxial crystal are composed of two shells, where *C*_1_ and *C*_2_ are the two corresponding optical axes, both within the *x*′–*z*′ plane. The two shells intersect at four singular points also known as diabolic points, where each optical axis connects the two opposite diabolic points passing through the origin of the momentum space. We emphasize that diabolic points only occurs in the *k*_*x*′_–*k*_*z*′_ plane because the value of *ɛ*_2_ is between *ɛ*_1_ and *ɛ*_3_.

We may define a new coordinate system *x*–*y*–*z* through a coordinate rotation of *θ* along the original principle axis *y*′. The relative permittivity tensor after the rotation can be written as

(1) $$\begin{array}{ll} {\overset{↔}{\varepsilon }}_{r} & =\left(\begin{array}{ccc} \varepsilon_{3}\sin^{2}\theta +\varepsilon_{1}\cos^{2}\theta & 0 & \left(\varepsilon_{3}-\varepsilon_{1}\right)\sin\theta \cos\theta \\ 0 & \varepsilon_{2} & 0\\ \left(\varepsilon_{3}-\varepsilon_{1}\right)\sin\theta \cos\theta & 0 & \varepsilon_{1}\sin^{2}\theta +\varepsilon_{3}\cos^{2}\theta \end{array}\right)\\ & =\left(\begin{array}{ccc} \varepsilon_{xx} & 0 & \varepsilon_{xz}\\ 0 & \varepsilon_{yy} & 0\\ \varepsilon_{xz} & 0 & \varepsilon_{zz} \end{array}\right). \end{array}$$

We choose such a rotation to maintain the original two optical axes within the newly formed *x*–*z* plane which requires keeping *y*′ axis unchanged. If we consider light propagation in this newly formed *x*–*y* plane, the wave equation can be derived as

(2) $$\begin{array}{c} \left\{\frac{1}{2}\left[\hat{p}-\hat{A}\right]{\overset{↔}{m}}^{-1}\left[\hat{p}-\hat{A}\right]-{\hat{A}}_{0}+V_{0}{\hat{\sigma }}_{0}\right\}\left(\begin{array}{c} E_{z}\\ \eta_{0}H_{z} \end{array}\right)=0, \end{array}$$

where the detailed derivation can be found in Supplementary Note 1. Surprisingly Eq. (2) bears certain similarity to the wave equation of a non-relativistic spin-1/2 particle traveling in SU(2) non-Abelian gauge potentials [36], where $\hat{p}=-i{\hat{\sigma }}_{0}\partial_{i}e_{i},\left(i=x,y\right)$ is the canonical momentum operator with ${\hat{\sigma }}_{0}$ being the 2D identity matrix, and $\overset{↔}{m}=\frac{1}{2}\left(\begin{array}{cc} {\overset{↔}{\varepsilon }}_{T} & 0\\ 0 & {\overset{↔}{I}}_{2\times 2} \end{array}\right)$ imitates an effective anisotropic mass with ${\overset{↔}{\varepsilon }}_{T}=\left(\begin{array}{cc} \varepsilon_{xx} & 0\\ 0 & \varepsilon_{yy} \end{array}\right)$ and ${\overset{↔}{I}}_{2\times 2}$ represents the 2D identity matrix. In particular, we have

(3) $$\begin{array}{c} \hat{A}=-{\hat{\sigma }}_{1}k_{0}\frac{\varepsilon_{xz}e_{y}}{2} \end{array}$$

and

(4) $$\begin{array}{ll} {\hat{A}}_{0} & ={\hat{A}}_{0}^{3}{\hat{\sigma }}_{3}=k_{0}^{2}\left(\frac{\varepsilon_{zz}-1}{2}\right){\hat{\sigma }}_{3}-\frac{k_{0}^{2}}{8}\varepsilon_{xz}^{2}\left(3{\overset{↔}{I}}_{2\times 2}+{\overset{↔}{\varepsilon }}_{T}^{-1}\right){\hat{\sigma }}_{3}, \end{array}$$

viewed as non-Abelian vector and scalar potentials, respectively, and

(5) $$\begin{array}{c} V_{0}{\hat{\sigma }}_{0}=\frac{k_{0}^{2}}{8}\varepsilon_{xz}^{2}\left(3{\overset{↔}{I}}_{2\times 2}-{\overset{↔}{\varepsilon }}_{T}^{-1}\right){\hat{\sigma }}_{0}-k_{0}^{2}\frac{\varepsilon_{zz}+1}{2}{\hat{\sigma }}_{0} \end{array}$$

as an additional Abelian scalar potential.

The SU(2) non-Abelian gauge potentials can also induce non-Abelian magnetic field and non-Abelian electric field as expressed as $\hat{B}=\nabla \times \hat{A}-i\hat{A}\times \hat{A}$ and $\hat{E}=\nabla {\hat{A}}_{0}+i\left[{\hat{A}}_{0},\hat{A}\right]$ , respectively [17]. Given that we consider a homogeneous biaxial crystal, the ∇ operator associated terms shall disappear. Moreover, with only ${\hat{\sigma }}_{1}$ component displayed in $\hat{A}$ , we have $\hat{A}\times \hat{A}=0$ , which indicates that non-Abelian magnetic field cannot be induced here. On the contrary, as long as ${\hat{A}}_{0}^{3}\neq 0$ , the non-Abelian vector potential and non-Abelian scalar potential shall in principle have noncommutative components (in Section 5, we will show that under certain conditions they can be commutable), and a non-Abelian electric field is expected.

Similar to the Lorentz force, which is given by the product between the electric current and the field, a virtual non-Abelian Lorentz force associated with the non-Abelian electric field emerges and influences spin wave packet dynamics [37], bearing certain similarity to the ZB of Dirac equation. It is no wonder that in optics, the induced non-Abelian electric field can also stimulates the trembled trajectory of light with certain pseudospin during propagation, *aka* ZB effect of light. We have to emphasize that the starting point of the seemingly complicated non-Abelian gauge field setup is so simple: the rotation of any biaxial material along the principle axis with a middle-value permittivity. The obtained effect is also dependent on the rotation angle *θ*, as all the parameters in Eq. (2) are functions of *θ*. Thus, a real space operation on a biaxial material provides a simple but powerful tool to explore the non-Abelian electric field induced effect and have it manipulated by varying *θ*.

The concept of *Zitterbewegung* (ZB) was first introduced by Schrodinger in 1930, referring to the rapid oscillation of a free electron in vacuum described by Dirac equation [38], [39]. These trembling motions of electron arise from the superposition between the positive and negative energy states. Now it has been recognized that ZB is not unique to Dirac electrons, but a generic feature of wave packet dynamics in spinor systems with certain linear dispersion relations [40]. The excitation of orthogonal modes can be used to observe ZB effect thus various systems were suggested including semiconductor lattice [41], trapped ions [42], graphene [43], ultracold atoms [44] in electronic systems and 2D photonic crystals [45], [46], photonic microcavities [47], zero-index metamaterials [48], binary waveguides [49], [50], moving potentials [51] in photonic systems. Apart from the original understanding of Dirac dispersion, ZB of light also can arise from the emergent non-Abelian gauge fields [17], [20].

After the rotation of *θ* to form the new coordinate system, we can now cut the EFS of the original biaxial material along the newly constructed *k*_*x*_–*k*_*y*_ plane and the corresponding equifrequency contours (EFCs) is highlighted by the red lines in Figure 2(b). We enlarged the region near *k*_*y*_ = 0 and parabolic curves can be found as shown in Figure 2(c). A gap size between the two curves at *k*_*y*_ = 0 can be obtained by solving the corresponding dispersion relation of the biaxial material with a wave number difference

(6) $$\begin{array}{c} 2\sigma =k_{0}\left|\sqrt{\frac{\varepsilon_{1}\varepsilon_{3}}{\varepsilon_{3}\sin^{2}\theta +\varepsilon_{1}\cos^{2}\theta }}-\sqrt{\varepsilon_{2}}\right|. \end{array}$$

The corresponding eigenstates at *k*_*y*_ = 0 can also be obtained, with

(7) $$\begin{array}{c} \psi_{1}:E_{1}=\left(0,1,0\right),H_{1}=\left(0,0,\frac{k_{x}}{\omega \mu_{0}}\right) \end{array}$$

and

(8) $$\begin{array}{c} \psi_{2}:E_{2}=\left(\frac{\left(\varepsilon_{3}-\varepsilon_{1}\right)\cos\theta \sin\theta }{\varepsilon_{1}\cos^{2}\theta +\varepsilon_{3}\sin^{2}\theta },0,1\right),H_{2}=\left(0,-\frac{k_{x}}{\omega \mu_{0}},0\right). \end{array}$$

It is evident that *ψ*_1_ is TM (transverse magnetic) dominant, where the polarization of magnetic field is along the *z* axis, perpendicular to the plane of propagation. *ψ*_2_ is on the contrary, TE (transverse electric) dominant because it has *E*_*z*_ contribution. The incidence of pure TM/TE light will excite *ψ*_1_/*ψ*_2_ only and a mixed polarization incidence can induce both *ψ*_1_ and *ψ*_2_. A different mode profile implies that their possible “different sign of mass” in analogy to Dirac physics and given their different wave numbers, interference may occur when *ψ*_1_ and *ψ*_2_ are simultaneously excited. As a universal feature of ZB, the oscillation period of beams, caused by the interference of the two orthogonal eigenmode shall be *T* = 2*π*/2σ, similar to previous reported works. In Supplementary Note 2, we provide simulation results on how to selectively excite *ψ*_1_ and *ψ*_2_ and their relationship to the emergence of ZB oscillations. Emergent non-Abelian electric field in the system shall induce ZB effect with a mixed excitation of *ψ*_1_ and *ψ*_2_.

In addition, it is worth mentioning that when rotated to a special angle $\theta_{0}=\sin^{-1}\sqrt{\frac{\varepsilon_{1}\left(\varepsilon_{2}-\varepsilon_{3}\right)}{\varepsilon_{2}\left(\varepsilon_{1}-\varepsilon_{3}\right)}}$ , a band crossing emerges in the EFCs at *k*_*y*_ = 0, as shown by red lines in Figure 2(d) whose enlarged view can be found in Figure 2(e). *θ*_0_ is the angle between one of the optical axis and the principle axis *k*_*x*′_ (see Figure 2(a)) and its value can be obtained by calculating the dispersion relation. *ψ*_1_ and *ψ*_2_ are thus degenerate and thus no interference shall occur. Substituting the expression of *θ*_0_ into the expression of 2*σ* yields the same result. The relation between this special angle and Abelian gauge field will be discussed later.

## 3 Simulation results

**Figure 3:**
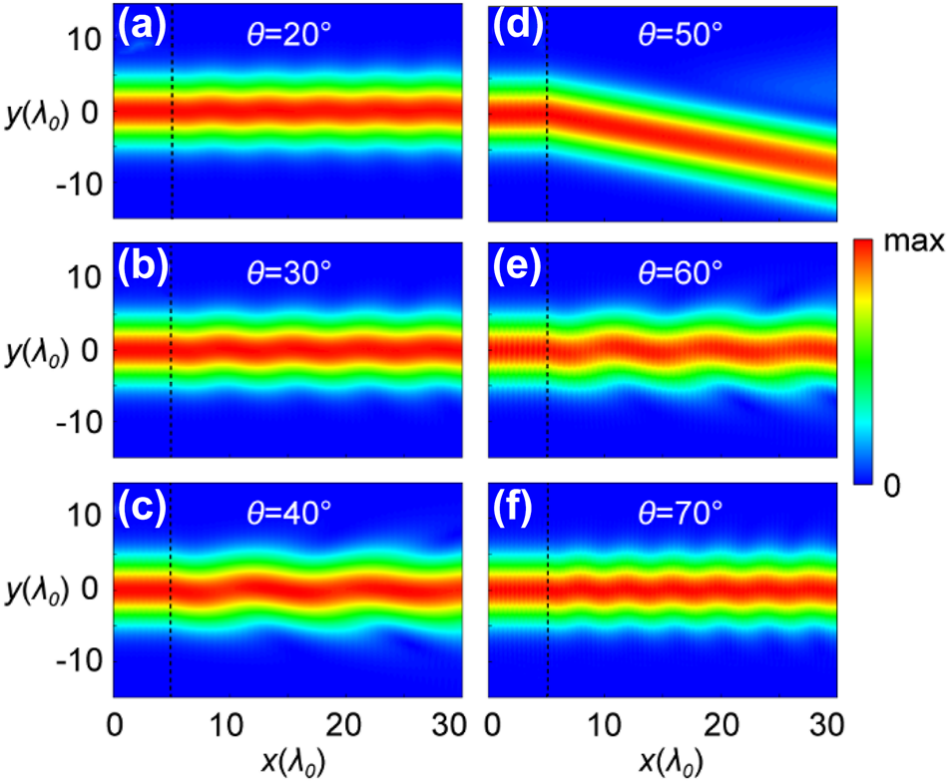
ZB effect in biaxial crystal. Distribution of field intensity when rotation angle *θ* = 20°, 30°, 40°, 50°, 60°, 70° within a biaxial material with *ɛ*_1_ = 2, *ɛ*_2_ = 1 and *ɛ*_3_ = 0.58. More examples of different material parameters can be found in Supplementary Notes.

Throughout this work, we use finite-element numerical package (COMSOL Multiphysics 5.5) to simulate corresponding field distributions. We start from a randomly chosen biaxial dielectrics with *ɛ*_1_ = 2, *ɛ*_2_ = 1 and *ɛ*_3_ = 0.58, and then rotate it by a series of angles from 20° to 70° to visualize the non-Abelian electric field induced ZB effect. A Gaussian-like beam with a beam width of *w* = 6*λ*_0_ (*λ*_0_ is the corresponding wavelength in vacuum) normally incident from background medium onto the rotated biaxial medium along positive *x* direction. The dielectric constant of background medium is adjusted to eliminate reflection as much as possible. The polarization of the incident beam is set as $E=\left(0,E_{0},E_{0}\right)$ where TE $\left(\psi_{2}\right)$ and TM $\left(\psi_{1}\right)$ modes are mixed with the *y* component of **E** excites *H*_*z*_ mode. For verification purpose, Figure 3(a)–(c) plot the *H*_*z*_ field intensity distributions while Figure 3(d)–(f) plot the *E*_*z*_ field intensity distributions. Trembling of the trajectory of beam center can be clearly observed.

The rotation angle *θ* also determines the oscillation period of the ZB effect. As shown in Figure 3(c) and (f), a period of 10.40*λ*_0_ (3.97*λ*_0_) is obtained from simulations which is in good agreement with the oscillation period $T=10.63\lambda_{0}\left(4.05\lambda_{0}\right)$ as predicted from Eq. (6). Using a different selection of rotation angle, we are capable of tuning the oscillation period. A *θ*_0_ = 50.28° is reached given the biaxial material with *ɛ*_1_ = 2, *ɛ*_2_ = 1, *ɛ*_3_ = 0.58 and thus it is no wonder that at *θ* = 50° (Figure 3(d)) no ZB oscillation emerges. From Figure 3, we can recognize that when *θ* is getting away from *θ*_0_, the period *T* decreases, indicating an increase of the wavenumber difference. Our numerical results are in perfect agreement with theoretical analysis.

Although the amplitude of ZB oscillation is not derived theoretically, we do observe some amplitude changes in Figure 3. As the trembling motion is induced by the interference of the two eigenmodes, their relative weight is decisive to the ZB amplitude. As the two eigenmodes *ψ*_1_ and *ψ*_2_ are also *θ* dependent, the fixed polarization used will change the relative weight between *ψ*_1_ and *ψ*_2_ and thus affect the ZB amplitude.

## 4 Experimental observation

**Figure 4:**
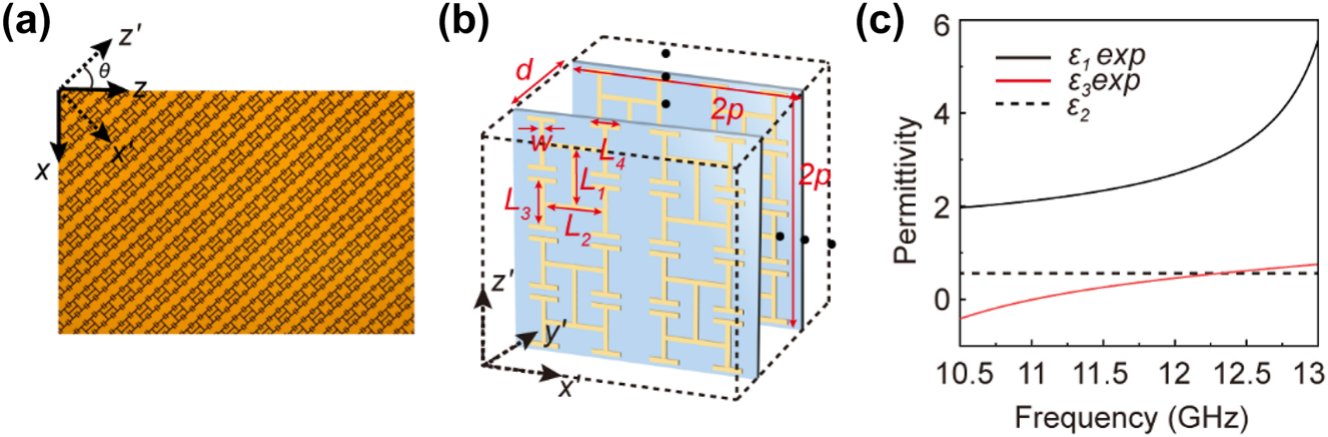
Biaxial metamaterial design. (a) Fractal metamaterial used in experiment. (b) Detailed metamaterial design with *p* = 5 mm, *d* = 6.5 mm, *L*_1_ = 2.3 mm, *L*_2_ = 2.4 mm, *L*_3_ = 1.8 mm, *L*_4_ = 1.2 mm, and copper width *w* = 0.2 mm. (c) Retrieved effective permittivity *ɛ*_1_ and *ɛ*_3_ (solid lines).

A microwave demonstration to the non-Abelian electric field induced ZB effect is conducted and the picture of experimental setup can be found in Figure 1(a). The tilted horn antenna is used to excite electromagnetic field with both *E*_*z*_ and *E*_*y*_ components, which exactly reflects the simulation setup discussed in the previous session. A microwave metamaterial design composed by multiple printed circuit boards (PCBs) with biaxial dielectric constants is used and a close-up top view of one piece of fabricated PCB can be found in Figure 4(a). Periodic array of copper fractal structures is printed on FR4 PCB with thickness 0.115 mm and relative permittivity 3.3. In order to obtain a tilted permittivity tensor, the fractal structure originally assembled in the *x*′*–* *z*′ plane is tilted by 45° to form the new *x–z* plane and thus the rotation angle *θ* = 45° is used to facilitate experimental measurement and sample fabrication. The periodicity *p* = 5 mm in *x*′*–* *z*′ plane is designed for the copper fractal structure, where other geometric parameters *L*_1_ = 2.3 mm, *L*_2_ = 2.4 mm, *L*_3_ = 1.8 mm, *L*_4_ = 1.2 mm, and copper width *w* = 0.2 mm as shown in Figure 4(b). In order to measure the electric field inside the biaxial metamaterial, a spacing of *d* = 6.5 mm is considered between every two PCBs along *y* direction, where in total 41 PCBs are used. Threads are used to fix the PCB metamaterial sample to two home-made acrylic stands and the spacing between neighboring PCBs is measured to be 6.5 ± 0.2 mm. The cross-section along the *y–z* plane of the sample is 260 mm × 212.1 mm, large enough to cover the beam width of the Gaussian-like incidence microwave beam, emitted from the horn antenna through a home-made acrylic lens. The length of the sample along *x* direction is 403 mm, long enough to accommodate a couple of ZB oscillations.

The effective permittivities *ɛ*_1_ and *ɛ*_3_ between 10.5 and 13 GHz is measured using its corresponding *S*-parameters. *ɛ*_2_ = 1.01 because of the geometrical average of permittivity. At different frequencies, the permittivity tensor yields different values. In other words, even though we consider one rotation angle in the experiment, the different biaxial material properties at different frequencies guarantee the generality of our recipe to visualize non-Abelian electric field induced ZB effect.

**Figure 5:**
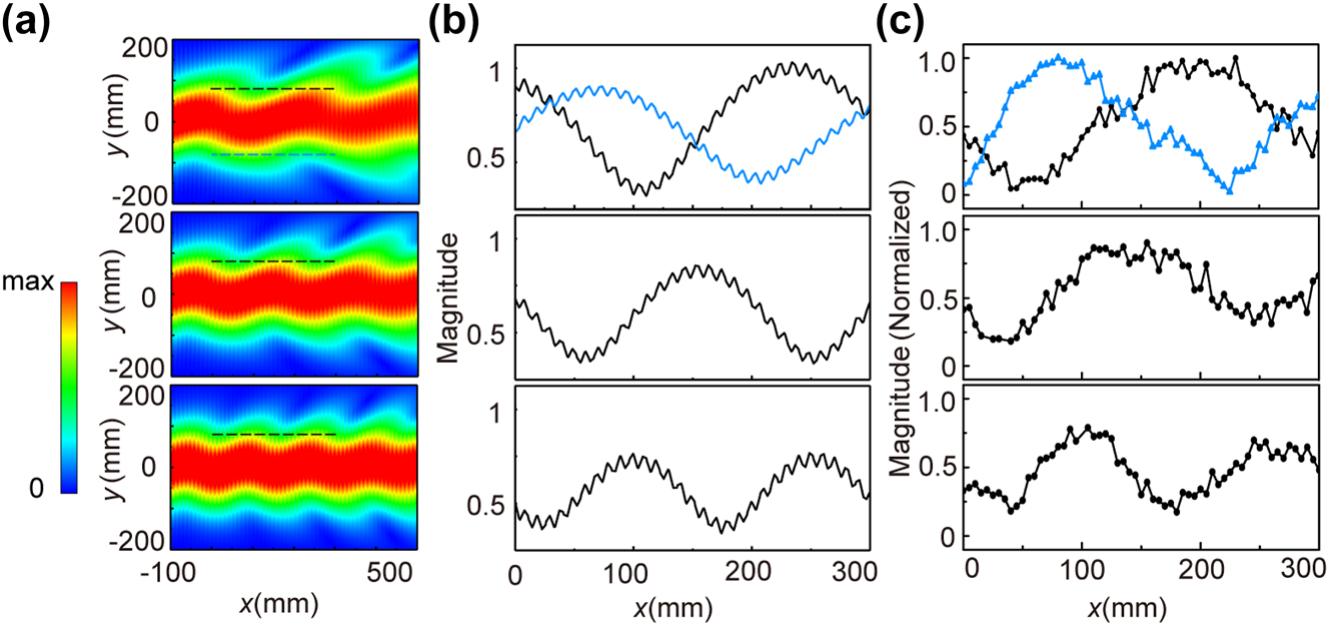
Experimental observation of ZB effect. (a) Simulation results of ZB effect using retrieved parameters from Figure 4(c) at 12.675 GHz, 12.800 GHz and 12.950 GHz (from top to bottom). (b) Field distribution along the dashed lines in (a). (c) Experiment results at the same positions of lines in (a).

Before the experimental measurements, numerical simulations are conducted. As can be seen in Figure 5(a), ZB effect is very obvious for the three frequencies: 12.675 GHz (top panel), 12.800 GHz (middle panel) and 12.950 GHz (bottom panel). The simulation results at 12.900 GHz for a longer *x*-direction span can be found in Figure 1(b). In the simulations, we adopt the retrieved dispersive material property as in Figure 4(c) and rotate the permittivity tensor by 45° and apply it to the whole simulation region. A Gaussian beam incidence with a beam width *w* = 6*λ*_0_ at *x* = −100 mm is applied whose beam center is located at *y* = 0 mm. At *x* = 500 mm, perfected matched layer (PML) conditions is applied to eliminate unwanted scattering. Obvious beam oscillation emerges and in the *E*_*z*_ distributions, the corresponding oscillation period matches with the *σ* value deducted from EFCs calculation from material properties. We may recover more details from the simulated results along the dotted lines. As PML boundary is set to match to vacuum but not to biaxial material, inevitable reflection occurs which explains the minute subwavelength oscillation on the line plots in Figure 5(b). In the top panel of Figure 5(b), two lines are plotted which located on the opposite sides of the beam center. The intensity changing along the propagation direction doesn’t necessarily indicate that the beam is oscillating along the direction perpendicular to the propagating direction: reflection induced intensity change will also do. However, the opposite trend of oscillation along the two lines at *y* = 80 mm (black) and *y* = −80 mm (azure) provides an unambiguous evidence that the beam center is oscillating. With the understanding of field and line distributions in Figure 5(a) and (b), the experimental results shown in Figure 5(c) can be the direct evidence to the non-Abelian electric field induced ZB effect. A dipole antenna polarized along the *z* direction was inserted into the spacing between neighboring PCBs to measure local *E*_*z*_ field distribution along *x* direction. Spatial resolution of 5 mm is used in the measurement. The actual metamaterial sample was located from $\left[-100\;\text{mm},\;303\;\text{mm}\right]$ and we measured the latter part of the sample. Two characteristic lines were taken into consideration, at *y* = 75 mm and *y* = −75 mm (between the 10th/11th and 32nd/33rd PCB boards). In the top panel of Figure 5(c), different trends of line oscillation precisely recover our argument of a beam center oscillation, *aka* the ZB effect, as well as the minute oscillation due to the impedance mismatch between our metamaterial to air. Similar oscillation period occurs indicating the good consistence between simulations and experiments, not only the ZB effect but also the retrieved effective material properties. Similar oscillation can also be observed at other frequencies where the material property meets the theoretical condition *ɛ*_1_ > *ɛ*_2_ > *ɛ*_3_. However, at the frequencies that this condition is not satisfied, the beam oscillation disappears while the minute reflection still exist.

## 5 Discussions and conclusions

### 5.1 ZB effect in natural material

**Figure 6:**
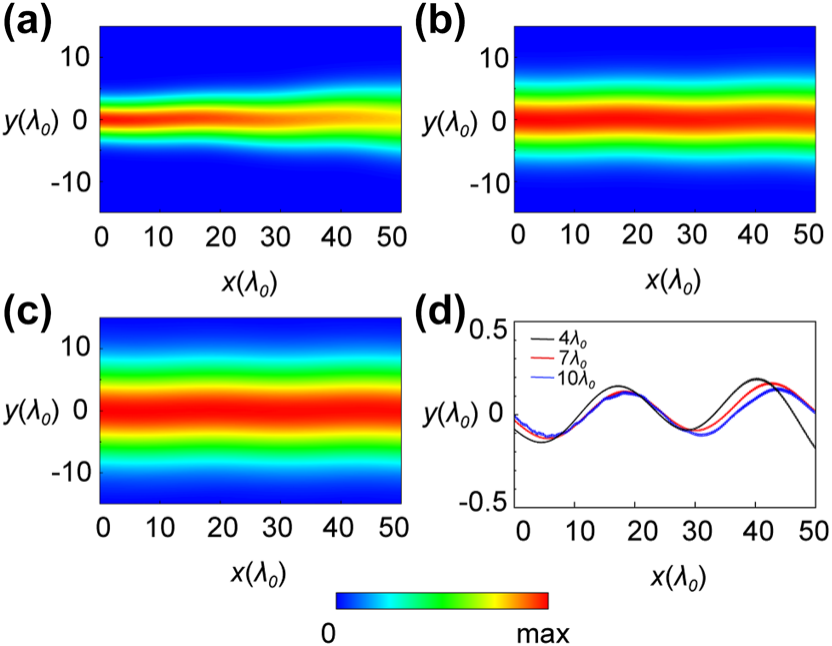
ZB effect in natural KTP crystal. (a)–(c) Non-Abelian electric field induced ZB effect in KTP crystal with *n*_1_ = 1.8648, *n*_2_ = 1.7712, and *n*_3_ = 1.7619 at *λ*_0_ = 632.8 nm and *θ* = 45° with incident beam widths *w* = 4, 7 and 10*λ*_0_; (d) the beam center oscillation amplitude at different incident beam widths.

Although the experiment is conducted at microwave frequencies using PCB metamaterials, the electric field induced ZB effect can also be observed at optical frequencies using natural biaxial crystals. We conduct simulation verification based on potassium titanyl phosphate (KTP) crystal [52], a biaxial crystal with biaxial refractive indices (*n*_1_ = 1.8648, *n*_2_ = 1.7712, and *n*_3_ = 1.7619 at *λ*_0_ = 632.8 nm), whose corresponding *θ*_0_ = 18.20° is retrieved. To facilitate future optical experiments, we still choose to rotate the crystal by 45° along the principal *y*′ axis and in Figure 6(a) we show a similar beam oscillation occurring in this natural material. The oscillation amplitude is relatively small compared to metamaterial scenarios due to the minute differences in refractive indices. In Figure 6(a)–(c), we find that ZB oscillation is not altered by incident beam width where the corresponding beam center oscillations are plotted in Figure 6(d). The achieved ZB period is 24.5*λ*_0_ close to the value of 25.02*λ*_0_ from theoretical derivation. The amplitude of ZB is about 0.2*λ*_0_ for all beam widths though the tightly focused beam with *w* = 4*λ*_0_ eventually diverges during propagation. The effect, though not as prominent as in microwave regime, shall still be apparent under an optical microscope.

### 5.2 Abelian and non-Abelian gauge field

The continuous rotation of biaxial media in real space offers a simple and elegant way to explore both Abelian and non-Abelian gauge fields. We have demonstrated that an arbitrary biaxial medium with any rotation angle along a specific principle axis can be used to observe non-Abelian electric field and its induced ZB effect. We may also define another special angle $\theta_{d}=\sin^{-1}\sqrt{\frac{\varepsilon_{1}-\varepsilon_{2}}{\varepsilon_{1}-\varepsilon_{3}}}$ . When turning the rotation angle *θ* = *θ*_*d*_, we have ${\overset{↔}{\varepsilon }}_{T}=\varepsilon_{2}{\overset{↔}{I}}_{2\times 2}$ , where in-plane duality symmetry is thus reserved. The non-Abelian scalar potential ${\hat{A}}_{0}$ can then be further simplified, where Eq. (4) becomes ${\hat{A}}_{0}={\hat{A}}_{0}^{3}{\hat{\sigma }}_{3}=k_{0}^{2}\frac{\varepsilon_{1}\varepsilon_{3}-\varepsilon_{2}^{2}}{2\varepsilon_{2}}{\hat{\sigma }}_{3}$ . Thus the value of the corresponding non-Abelian electric field can be obtained as $\hat{E}=i\left[{\hat{A}}_{0},\hat{A}\right]=k_{0}^{3}\frac{\varepsilon_{xz}\left(\varepsilon_{1}\varepsilon_{3}-\varepsilon_{2}^{2}\right)}{2\sqrt{\varepsilon_{2}^{3}}}{\hat{\sigma }}_{2}e_{y}$ , which is in consistent with Ref. [17]. Furthermore, if a strong constraint *θ*_*d*_ = *θ*_0_ can be satisfied, which means $\varepsilon_{1}\varepsilon_{3}-\varepsilon_{2}^{2}=0$ , the non-Abelian electric field induced ZB effect will disappear since the non-Abelian electric field itself vanishes. An optical beam splitting emerges because of the remaining vector potential, which is the exact scenario discussed in Ref. [53]. Consequently, the non-Abelian gauge field metamaterial is thus reduced to Abelian metamaterial. Detailed derivation and simulation results can be found in Supplementary Note 3.

ZB effect has been used to demonstrate different peculiar properties predicted by Dirac equations, where in this work, visualizing ZB effect offers an approach to distinguish non-Abelian media from Abelian media. In the Abelian gauge field metamaterials, a particular type of tilted anisotropy in the constitutive parameters provide a vector gauge potential which split the originally degenerated dispersion curves but still exhibit one degeneracy point. In the non-Abelian scenario, a different type of tilted anisotropy offers additional gauge potentials which do not commute with each other. Thus, the degenerate point in the Abelian scenario is lifted, enabling the unique ZB effect. We shall also emphasize that different from ZB effects previously demonstrated relying on latticed-structures [44], [45], [46], [47], [49], [50], the non-Abelian electric field induced ZB effect occurs even in a homogeneous natural material. The oscillation reported here also occurs in real space rather than in frequency or time domains [45], [46], where a direct visualization of ZB effect is enabled.

In summary, we extend the previous research efforts of gauge field materials to an extremely simple level that non-Abelian electric field can be induced by a real-space rotation of any biaxial material. Trembling motion of an incident optical beam, *aka* optical ZB effect, can be visualized within an appropriate selection of light propagation plane which is a direct consequence of the non-Abelian electric field. A microwave metamaterial is designed and fabricated where unambiguous evidence of beam oscillation is achieved with excellent agreement with theoretical analysis and numerical simulations. In contrast with other gauge fields in anisotropic materials constrained by the condition of in-plane duality symmetry, we find that non-Abelian physics is also general in optics where the real space operation of materials can directly manipulate the associated non-Abelian properties. The successful observation of real-space ZB effect induced by the non-Abelian electric field provides not only another example that light beam can propagate along curved path even in homogeneous media but also simulates the exploration of real-space gauge fields optics.

## Supplementary Material

Supplementary Material Details
