# Supplementary material for: A general recipe to observe non-Abelian gauge field in metamaterials
Source: Nanophotonics. 2024 Oct 30;14(8):1135–43. doi: 10.1515/nanoph-2024-0414 (PMC12019942; doi:10.1515/nanoph-2024-0414)
Supplement: Supplementary file 1 — Supplementary Material Details [file j_nanoph-2024-0414_suppl_001.docx]

**Supplementary Note**

1. **Derivation of wave equation with effective non-Abelian gauge field**

Following the scheme discussed in the main text, we have a biaxial dielectric material with relative permittivity tensor and permeability tensor as $\left( \begin{matrix} \varepsilon_{1} & 0 & 0 \\ 0 & \varepsilon_{2} & 0 \\ 0 & 0 & \varepsilon_{3} \end{matrix} \right)$ and $\mu_{r}=I_{3\times3}$ ($I_{3\times3}$ is the $3\times3$ identity matrix), respectively in the principle coordinate system $x’$-$y’$-$z’$. By rotating it along the $y'$ axis (also the new $y$ axis) for an angle $\theta$, we obtain the new permittivity tensor in the new $x$-$y$-$z$ coordinate system

$$\begin{aligned} \varepsilon_{r}=\left( \begin{matrix} \varepsilon_{xx} & 0 & \varepsilon_{xz} \\ 0 & \varepsilon_{yy} & 0 \\ \varepsilon_{xz} & 0 & \varepsilon_{zz} \end{matrix} \right),\#\left( S1 \right) \end{aligned}$$

where $\varepsilon_{xx}=\varepsilon_{3}\sin^{2} \theta+\varepsilon_{1}\cos^{2} \theta,$ $\varepsilon_{xz}=\left( \varepsilon_{3}-\varepsilon_{1} \right) \sin\theta\cos\theta,$ $\varepsilon_{yy}=\varepsilon_{2}$ , $\varepsilon_{zz}=\varepsilon_{1}\sin^{2} \theta+\varepsilon_{3}\cos^{2} \theta.$ Here, without loss of generality, we assume that $\varepsilon_{1}>\varepsilon_{2}>\varepsilon_{3}$. Eq. (S1) can be decomposed into $x$*-*$y$ plane and out-of-plane components

$$\begin{aligned} \varepsilon_{r}=\left( \begin{matrix} \varepsilon_{T} & \mathbf{g} \\ \mathbf{g}^{\dagger} & \varepsilon_{zz} \end{matrix} \right),\#\left( S2 \right) \end{aligned}$$

where$\varepsilon_{T}=\left( \begin{matrix} \varepsilon_{xx} & 0 \\ 0 & \varepsilon_{yy} \end{matrix} \right)$, $\mathbf{g=}\left( \begin{matrix} \varepsilon_{xz} \\ 0 \end{matrix} \right)=\varepsilon_{xz} \mathbf{e}_{x}$ , where $\mathbf{e}_{x}$ is the unit vector in the $x$ direction.

We thus can rewrite Maxwell equations considering in-plane and out-of-plane waves separately, and yield

$$\begin{aligned} \left( \begin{matrix} \nabla_{T}\times& 0 \\ {ik}_{0}\tilde{\mathbf{g}}\times& \nabla_{T}\times\end{matrix} \right)\left( \begin{matrix} \mathbf{E}_{z} \\ \eta_{0}\mathbf{H}_{z} \end{matrix} \right)=\left( \begin{matrix} 0 & ik_{0}I_{2\times2} \\ -ik_{0}\varepsilon_{T} & 0 \end{matrix} \right)\left( \begin{matrix} \mathbf{E}_{T} \\ \eta_{0}\mathbf{H}_{T} \end{matrix} \right),\#\left( S3a \right) \end{aligned}$$

$$\begin{aligned} \left( \begin{matrix} \nabla_{T}\times& 0 \\ {-ik}_{0}{\tilde{\mathbf{g}}}^{\dagger}\times& \nabla_{T}\times\end{matrix} \right)\left( \begin{matrix} \mathbf{E}_{T} \\ \eta_{0}\mathbf{H}_{T} \end{matrix} \right)=\left( \begin{matrix} 0 & ik_{0} \\ -ik_{0}\varepsilon_{zz} & 0 \end{matrix} \right)\left( \begin{matrix} \mathbf{E}_{z} \\ \eta_{0}\mathbf{H}_{z} \end{matrix} \right),\#\left( S3b \right) \end{aligned}$$

where $\tilde{\mathbf{g}}\boldsymbol{=}\mathbf{e}_{z}\boldsymbol{\times}\mathbf{g}$, $\eta_{0}=\sqrt{\mu_{0}/\varepsilon_{0}}$.

Eq. (S3) can be further rewritten as

$$\begin{aligned} \left[ \hat{\sigma}_{0} \left( \nabla_{T}\times\right)+\hat{\sigma}_{1} \left( {ik}_{0}\frac{\tilde{\mathbf{g}}\times}{2} \right)-i\hat{\sigma}_{2} \left( {ik}_{0}\frac{\tilde{\mathbf{g}}\times}{2} \right) \right]\left( \begin{matrix} \mathbf{E}_{z} \\ \eta_{0}\mathbf{H}_{z} \end{matrix} \right)={-k}_{0}\left( \begin{matrix} I_{2\times2} & 0 \\ 0 & \varepsilon_{T} \end{matrix} \right)\hat{\sigma}_{2}\left( \begin{matrix} \mathbf{E}_{T} \\ \eta_{0}\mathbf{H}_{T} \end{matrix} \right),\#\left( S4a \right) \end{aligned}$$

$$\begin{aligned} \left[ \hat{\sigma}_{0} \left( \nabla_{T}\times\right)-\hat{\sigma}_{1} \left( {ik}_{0}\frac{{\tilde{\mathbf{g}}}^{\dagger}\times}{2} \right)+i\hat{\sigma}_{2} \left( {ik}_{0}\frac{{\tilde{\mathbf{g}}}^{\dagger}\times}{2} \right) \right]\left( \begin{matrix} \mathbf{E}_{T} \\ \eta_{0}\mathbf{H}_{T} \end{matrix} \right)=\left[ \hat{\sigma}_{1} \left( {ik}_{0}\frac{1-\varepsilon_{zz}}{2} \right)+i\hat{\sigma}_{2} \left( {ik}_{0}\frac{1+\varepsilon_{zz}}{2} \right) \right]\left( \begin{matrix} \mathbf{E}_{z} \\ \eta_{0}\mathbf{H}_{z} \end{matrix} \right),\#\left( S4b \right) \end{aligned}$$

where $\hat{\sigma}_{0}=\left( \begin{matrix} 1 & 0 \\ 0 & 1 \end{matrix} \right), \hat{\sigma}_{1}=\left( \begin{matrix} 0 & 1 \\ 1 & 0 \end{matrix} \right), \hat{\sigma}_{2}=\left( \begin{matrix} 0 & -i \\ i & 0 \end{matrix} \right), \hat{\sigma}_{3}=\left( \begin{matrix} 1 & 0 \\ 0 & -1 \end{matrix} \right)$ are Pauli matrices.

Multiply $\hat{\sigma}_{2}\left( \begin{matrix} I_{2\times2} & 0 \\ 0 & \varepsilon_{T} \end{matrix} \right)^{-1}$ to Eq. (S4a) and substitute it into the multiplication of $(-\hat{\sigma}_{2}k_{0})$ to Eq. (S4b), we shall eliminate $\mathbf{E}_{T}$ and $\mathbf{H}_{T}$ to obtain

$$\begin{aligned} \begin{aligned} \left\{ \left[ \hat{\sigma}_{0} \left( \nabla_{T}\times\right)+\hat{\sigma}_{1} \left( {ik}_{0}\frac{{\tilde{\mathbf{g}}}^{\dagger}\times}{2} \right)+i\hat{\sigma}_{2} \left( {ik}_{0}\frac{{\tilde{\mathbf{g}}}^{\dagger}\times}{2} \right) \right]\left( \begin{matrix} I_{2\times2} & 0 \\ 0 & \varepsilon_{T} \end{matrix} \right)^{-1}\left[ \hat{\sigma}_{0} \left( \nabla_{T}\times\right)+\hat{\sigma}_{1} \left( {ik}_{0}\frac{\tilde{\mathbf{g}}\times}{2} \right)-i\hat{\sigma}_{2} \left( {ik}_{0}\frac{\tilde{\mathbf{g}}\times}{2} \right) \right] \right. \\ \left. -k_{0}^{2}\left[ \hat{\sigma}_{3} \left( \frac{\varepsilon_{zz}-1}{2} \right)+\hat{\sigma}_{0} \left( \frac{\varepsilon_{zz}+1}{2} \right) \right] \right\}\left( \begin{matrix} \mathbf{E}_{z} \\ \eta_{0}\mathbf{H}_{z} \end{matrix} \right)=0. \end{aligned}\#\left( S5 \right) \end{aligned}$$

After expanding Eq. (S5), we obtain

$$\begin{aligned} \begin{aligned} \left\{ \frac{1}{2}\left[ \hat{\mathbf{p}}-\hat{\sigma}_{1}k_{0}\frac{\mathbf{g}\times\mathbf{e}_{z}}{2}-{i\hat{\sigma}}_{2}k_{0}\frac{\mathbf{g}\times\mathbf{e}_{z}}{2} \right]m^{-1}\left[ \hat{\mathbf{p}}-\hat{\sigma}_{1}k_{0}\frac{\mathbf{g}\times\mathbf{e}_{z}}{2}+i\hat{\sigma}_{2}k_{0}\frac{\mathbf{g}\times\mathbf{e}_{z}}{2} \right] \right. \\ \left. -k_{0}^{2}\left[ \hat{\sigma}_{3} \left( \frac{\varepsilon_{zz}-1}{2} \right)+\hat{\sigma}_{0} \left( \frac{\varepsilon_{zz}+1}{2} \right) \right] \right\}\left( \begin{matrix} E_{z} \\ \eta_{0}H_{z} \end{matrix} \right)=0, \end{aligned}\#\left( S6 \right) \end{aligned}$$

where $\hat{\mathbf{p}}=-i\hat{\sigma}_{0} \partial_{i}\mathbf{e}_{i},\left( i=x,y \right)$, $m^{-1}=2\left( \begin{matrix} \varepsilon_{T}^{-1} & 0 \\ 0 & I_{2\times2} \end{matrix} \right)$.

Eq. (S6) can be further simplified as

$$\begin{aligned} \left\{ \frac{1}{2}\left[ \hat{\mathbf{p}}-\hat{A}+i\hat{A}_{I} \right]m^{-1}\left[ \hat{\mathbf{p}}-\hat{A}-i\hat{A}_{I} \right]-k_{0}^{2}\left[ \hat{\sigma}_{3} \left( \frac{\varepsilon_{zz}-1}{2} \right)+\hat{\sigma}_{0} \left( \frac{\varepsilon_{zz}+1}{2} \right) \right] \right\}\left( \begin{matrix} E_{z} \\ \eta_{0}H_{z} \end{matrix} \right)=0,\#\left( S7 \right) \end{aligned}$$

by substituting $\hat{A}=\hat{\sigma}_{1}k_{0}\frac{\mathbf{g}\times\mathbf{e}_{z}}{2}$, $\hat{A}_{I}={-\hat{\sigma}}_{2}k_{0}\frac{\mathbf{g}\times\mathbf{e}_{z}}{2}$.

In Eq. (S7), by taking $i\hat{A}_{I}$ out of the bracket and after same mathematical operations, we obtain

$$\begin{aligned} \begin{aligned} \left\{ \frac{1}{2}\left[ \hat{\mathbf{p}}-\hat{A} \right]m^{-1}\left[ \hat{\mathbf{p}}-\hat{A} \right]+\frac{k_{0}^{2}}{8}\varepsilon_{xz}^{2}[\hat{\sigma}_{0}\left( 3I_{2\times2}-\varepsilon_{T}^{-1} \right)+\hat{\sigma}_{3}(3I_{2\times2}+\varepsilon_{T}^{-1})] \right. \\ \left. -k_{0}^{2}\left[ \hat{\sigma}_{3} \left( \frac{\varepsilon_{zz}-1}{2} \right)+\hat{\sigma}_{0} \left( \frac{\varepsilon_{zz}+1}{2} \right) \right] \right\}\left( \begin{matrix} E_{z} \\ \eta_{0}H_{z} \end{matrix} \right)=0. \end{aligned}\#\left( S8 \right) \end{aligned}$$

Eq. (S8) can be further simplified to a more commonly recognizable form as

$$\begin{aligned} \left\{ \frac{1}{2}\left[ \hat{\mathbf{p}}-\hat{A} \right]m^{-1}\left[ \hat{\mathbf{p}}-\hat{A} \right]-\hat{A}_{0}+V_{0}\hat{\sigma}_{0} \right\}\left( \begin{matrix} E_{z} \\ \eta_{0}H_{z} \end{matrix} \right)=0,\#\left( S9 \right) \end{aligned}$$

where we substitute

$$\begin{aligned} \hat{A}=\hat{\sigma}_{1}k_{0}\frac{\mathbf{g}\times\mathbf{e}_{\boldsymbol{z}}}{2}=-\hat{\sigma}_{1}k_{0}\frac{\varepsilon_{xz}\mathbf{e}_{\boldsymbol{y}}}{2},\#\left( S10 \right) \end{aligned}$$

$$\begin{aligned} \hat{A}_{0}=\hat{A}_{0}^{3}\hat{\sigma}_{3}=k_{0}^{2} \hat{\sigma}_{3}\left( \frac{\varepsilon_{zz}-1}{2} \right)-\frac{k_{0}^{2}}{8}\varepsilon_{xz}^{2}\hat{\sigma}_{3}\left( 3I_{2\times2}+\varepsilon_{T}^{-1} \right),\#\left( S11 \right) \end{aligned}$$

$$\begin{aligned} V_{0}\hat{\sigma}_{0}=\frac{k_{0}^{2}}{8}\varepsilon_{xz}^{2}\left( 3I_{2\times2}-\varepsilon_{T}^{-1} \right)\hat{\sigma}_{0}-k_{0}^{2}\frac{\varepsilon_{zz}+1}{2}\hat{\sigma}_{0}.\#\left( S12 \right) \end{aligned}$$

Following the analysis in Ref. [S1], $\hat{A}$ is non-Abelian vector potential, $\hat{A}_{0}$ is non-Abelian scalar potential, $V_{0}$ is Abelian scalar potential, and the associated non-Abelian magnetic field and non-Abelian electric field are $\hat{\mathcal{B}}=\nabla\times\hat{A}-i \hat{A}\times\hat{A}$ and $\hat{\mathcal{E}}=\nabla\hat{A}_{0}+i\left[ \hat{A}_{0},\hat{A} \right]$, respectively. Since the biaxial crystal under consideration is homogeneous, the $\nabla$ operator associated terms shall disappear. Moreover, with only $\hat{\sigma}_{1}$ component in $\hat{A}$, $\hat{A}\times\hat{A}=0$, which yields a vanishing non-Abelian magnetic field. **The non-Abelian gauge field existing here can only be non-Abelian electric field.** If $\hat{A}_{0}^{3}\neq0$, the non-Abelian vector potential and non-Abelian scalar potential shall in principle have noncommutative components, and thus nonzero non-Abelian electric field emerges, enabling a *Zitterbewegung* (ZB)-like trembling optical beam propagation as discussed and observed in the main text.

1. **Eigenstates analysis at** $\boldsymbol{k}_{\boldsymbol{y}}\boldsymbol{=0}$ **in the *x-y* plane**

Provided the rotated permittivity tensor as

$$\begin{aligned} \varepsilon_{r}=\left( \begin{matrix} \varepsilon_{3}\sin^{2} \theta+\varepsilon_{1}\cos^{2} \theta& 0 & \left( \varepsilon_{3}-\varepsilon_{1} \right) \sin\theta\cos\theta\\ 0 & \varepsilon_{2} & 0 \\ \left( \varepsilon_{3}-\varepsilon_{1} \right) \sin\theta\cos\theta& 0 & \varepsilon_{1}\sin^{2} \theta+\varepsilon_{3}\cos^{2} \theta\end{matrix} \right)\#\left( S13 \right) \end{aligned}$$

under consideration, we may obtain the dispersion relations in the $x$*-*$y$ plane. With $k_{y}=0$, two solutions of $k_{x}$, the wavenumber along $x$ direction, are obtained as

$$\begin{aligned} k_{x}=k_{0}\sqrt{\varepsilon_{2}},\#\left( S14 \right) \end{aligned}$$

and

$$\begin{aligned} k_{x}=k_{0}\sqrt{\frac{\varepsilon_{1}\varepsilon_{3}}{\varepsilon_{3}\sin^{2} \theta+\varepsilon_{1}\cos^{2} \theta}}.\#\left( S15 \right) \end{aligned}$$

Their difference yields

$$\begin{aligned} 2\sigma=k_{0}\left| \sqrt{\frac{\varepsilon_{1} \varepsilon_{3}}{\varepsilon_{3}\sin^{2} \theta+\varepsilon_{1}\cos^{2} \theta}}-\sqrt{\varepsilon_{2}} \right|.\#\left( S16 \right) \end{aligned}$$

The eigenmode $\psi_{1}$ can be achieved by inserting Eq. (S14) into the wave equation and we reach

$$\begin{aligned} {\psi_{1}:\mathbf{E}}_{1}=\left( 0,1,0 \right),\mathbf{H}_{1}=\left( 0,0,\frac{k_{x}}{\omega\mu_{0}} \right),\#\left( S17 \right) \end{aligned}$$

which is TM (transverse magnetic) dominant, where the polarization of magnetic field is perpendicular to the plane of propagation. And similarly, the eigenmode $\psi_{2}$ is achieved as

$$\begin{aligned} {\psi_{2}:\mathbf{E}}_{2}=\left( \frac{\left( \varepsilon_{3}-\varepsilon_{1} \right)\cos\theta\sin\theta}{\varepsilon_{1}\cos^{2} \theta+\varepsilon_{3}\sin^{2} \theta},0,1 \right),\mathbf{H}_{2}=\left( 0,-\frac{k_{x}}{\omega\mu_{0}},0 \right).\#\left( S18 \right) \end{aligned}$$

The mode profile of $\psi_{2}$ is TE (transverse electric) dominant because the only out-of-plane mode exists is $E_{z}$ contribution.

As following, we will verify that the successful excitation of both eigenmodes are essential to induce ZB effects by numerical simulations. Consider an incident beam propagating along the positive $x$ direction with TM polarization, and thus only $\psi_{1}$ is excited. As shown in Figure S1(a), we only obtain a straight light propagation trajectory without beam oscillation. By changing the incident polarization to TE polarization, eigenmode $\psi_{2}$ is solely excited and a similar straight trajectory is obtained, as illustrated in Figure S1(b). By switching to an incident polarization with both $E_{y}$ and $E_{z}$ components as in Figure S1(c), we expect that both $\psi_{1}$ and $\psi_{2}$ modes are simultaneously excited and the emergence of trembling of optical beam is visualized. Such a polarization is exactly the polarization provided by the tilted horn antenna setup used in our microwave experiment, as shown in Figure 1(a) in the main text. Mixed mode excitation to induce interference is also important to the excitation of optical ZB effect as previously reported.


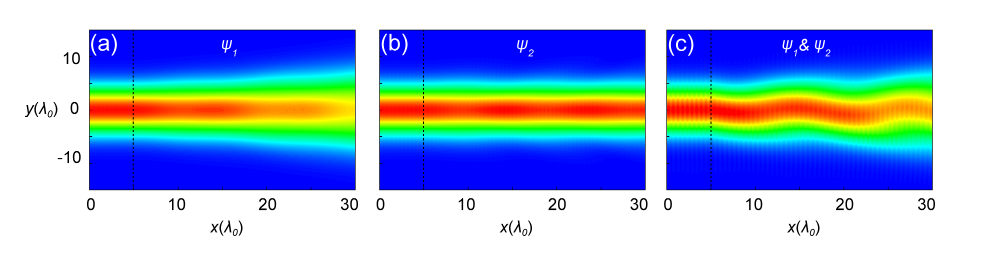


**Figure S1** Mixed polarization of orthogonal eigenmodes leads to ZB effect. (a/b) TM/TE polarization incidence. (c) Mixed TE and TM polarization incidence leads to the interference of two eigenmodes. Parameters of the biaxial material are given as $\varepsilon_{1}=2,\varepsilon_{2}=1$, and $\varepsilon_{3}=2/3$ with $\theta$=$35^{\circ}$.

1. **Abelian and non-Abelian gauge field in biaxial crystals**

The rotated permittivity tensor has been used to explore both Abelian and non-Abelian gauge fields [S1], [S2] with a necessary condition of in-plane duality symmetry. Here, starting from a more general arbitrary rotation of biaxial crystal, we still can reach previously reported results. The in-plane duality symmetry can be satisfied if a special rotation angle $\theta=\theta_{d}=\sin^{-1} \sqrt{\frac{\varepsilon_{2}-\varepsilon_{1}}{\varepsilon_{3}-\varepsilon_{1}}}$ is taken, where we have $\varepsilon_{xx}=\varepsilon_{yy}=\varepsilon_{2},$ $\varepsilon_{zz}=\varepsilon_{1}+\varepsilon_{3}-\varepsilon_{2},$ $\varepsilon_{xz}=\sqrt{\left( \varepsilon_{1}-\varepsilon_{2} \right)\left( \varepsilon_{2}-\varepsilon_{3} \right)}$. Consequently, in-plane duality is thus satisfied with $\varepsilon_{T}=\varepsilon_{2}I_{2\times2}$. By redefining $\varepsilon'=\left( \begin{matrix} \varepsilon_{1} & 0 & 0 \\ 0 & \varepsilon_{2} & 0 \\ 0 & 0 & \varepsilon_{3} \end{matrix} \right)/\varepsilon_{2}$,$\varepsilon_{0}^{'}=\varepsilon_{0}\varepsilon_{2}$, we have ${\varepsilon'}_{T}=I_{2\times2}$,, and substitute these parameters into Eq. (S10) and (S11), we obtain

$$\begin{aligned} \hat{A}=-\hat{\sigma}_{1}k_{0}^{'}\frac{\varepsilon_{xz}^{'}\mathbf{e}_{y}}{2}=-\hat{\sigma}_{1}k_{0}\frac{\varepsilon_{xz} \mathbf{e}_{y}}{2\sqrt{\varepsilon_{2}}},\#\left( S19 \right) \end{aligned}$$

$$\begin{aligned} \hat{A}_{0}=\hat{A}_{0}^{3}\hat{\sigma}_{3}=k_{0}^{'2}\hat{\sigma}_{3} \left( \frac{\varepsilon_{zz}^{'}-1}{2} \right)-\frac{1}{2}k_{0}^{'2}{\hat{\sigma}_{3}\varepsilon}_{xz}^{'2}=k_{0}^{2}\hat{\sigma}_{3}\frac{\varepsilon_{1}\varepsilon_{3}-\varepsilon_{2}^{2}}{2\varepsilon_{2}}.\#\left( S20 \right) \end{aligned}$$

Now with a clearer form of $\hat{A}_{0}$ and $\hat{A}$, the rigorous expression of non-Abelian electric field can be reached as

$$\begin{aligned} \hat{\mathcal{E}}=i\left[ \hat{A}_{0},\hat{A} \right]=k_{0}^{3} \frac{\varepsilon_{xz}\left( \varepsilon_{1}\varepsilon_{3}-\varepsilon_{2}^{2} \right)}{2\sqrt{\varepsilon_{2}^{3}}}\hat{\sigma}_{2}\mathbf{e}_{y}\boldsymbol{,}\#\left( S22 \right) \end{aligned}$$

which is consistent with Ref. [S1].

In Figure S2(a) and Figure 2(b), we plot the field distributions with mixed TE and TM polarization incidence to a biaxial material of $\varepsilon_{1}=2,\varepsilon_{2}=1$, and $\varepsilon_{3}=2/3$ and in the new plane with rotation angle $\theta$ equal to$55^{\circ}$ and $60^{\circ}$, respectively. The corresponding $\theta_{d}$ and $\theta_{0}$ of the biaxial material are $\theta_{d}$ =$60^{\circ}$ and $\theta_{0}$ =$45^{\circ}$. Hence when ${\theta=\theta}_{d}=60^{\circ}$, the in-plane duality symmetry is satisfied while when $\theta$=$55^{\circ}$, there exists no in-plane duality. However, in both cases, the ZB motion of light beam is obvious, indicating that the existence of non-Abelian electric field doesn’t necessarily requires in-plane duality.

Moreover, if we want a more straight condition with $\theta=\theta_{d}=\theta_{0}$, from $\theta_{d}=\sin^{-1} \sqrt{\frac{\varepsilon_{2}-\varepsilon_{1}}{\varepsilon_{3}-\varepsilon_{1}}}$ and $\theta_{0}=\sin^{-1} \sqrt{\frac{\varepsilon_{1}(\varepsilon_{2}-\varepsilon_{3})}{\varepsilon_{2}(\varepsilon_{1}-\varepsilon_{3})}}$, we shall reach $\varepsilon_{1}\varepsilon_{3}=\varepsilon_{2}^{2}$. By substituting it into Eq. (S19) and (S20), we shall have

$$\begin{aligned} \hat{A}=-\hat{\sigma}_{1}k_{0}\frac{\varepsilon_{xz} \mathbf{e}_{y}}{2\sqrt{\varepsilon_{2}}},\#\left( S23 \right) \end{aligned}$$

$$\begin{aligned} \hat{A}_{0}=k_{0}^{2}\hat{\sigma}_{3}\frac{\varepsilon_{1}\varepsilon_{3}-\varepsilon_{2}^{2}}{2\varepsilon_{2}}=0.\#\left( S24 \right) \end{aligned}$$

Consequently, $\hat{\mathcal{E}}=i\left[ \hat{A}_{0},\hat{A} \right]\equiv0$. The non-Abelian scalar potential and the non-Abelian electric field vanish though the non-Abelian vector potential persists. The non-Abelian gauge field reduces to Abelian gauge field, which is the case discussed in Ref. [S2]. Figures S2(c) and S2(d) plot the field distributions for $\varepsilon_{1}=4,\varepsilon_{2}=2$, and $\varepsilon_{3}=1$ with $\theta$ equal to $45^{\circ}$ and $54.74^{\circ}$, respectively. Both $\theta_{d}$ and $\theta_{0}$ of this biaxial crystal are equal to $54.74^{\circ}$. In Figure 2(c), without in-plane duality, we still observe the ZB effect. But in Figure S2(d), as $\theta=\theta_{d}=\theta_{0}$ is satisfied, beam splitting due to the existence of Abelian gauge field as reported in Ref. [S2] is observed.


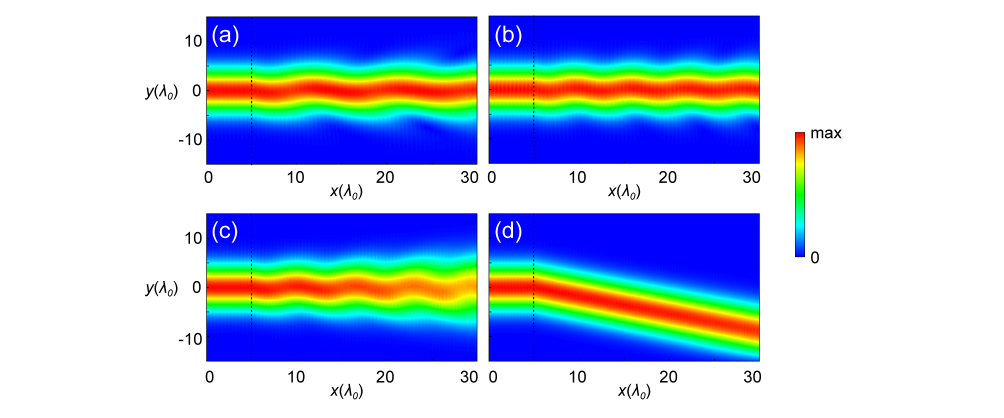


**Figure S2** Abelian and non-Abelian gauge field in biaxial crystals. (a, b) Non-Abelian electric field induces ZB effect in biaxial crystal with $\varepsilon_{1}=2,\varepsilon_{2}=1$,and $\varepsilon_{3}=2/3$. (a) $\theta$ = $55^{\circ}$ (b) $\theta$ = $60^{\circ}$. (c, d) Non-Abelian gauge field and Abelian gauge field in biaxial crystal with $\varepsilon_{1}=4,\varepsilon_{2}=2$, and $\varepsilon_{3}=1$. (c) $\theta$ = $45^{\circ}$ (d)$\theta=$ $54.74^{\circ}$.

1. **Amplitude of Zitterbewegung effect**

**
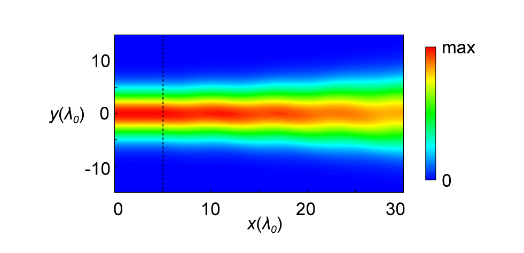
**

**Figure S3** ZB effect excited by $\mathbf{E}=(0,E_{0},{3E}_{0})$ in biaxial crystal with $\varepsilon_{1}=4,\varepsilon_{2}=2,\varepsilon_{3}=1$ and $\theta$ = $45^{\circ}$.

The equations of motion for spin $\mathbf{s}$ of a classical relativistic particle coupled to the non-Abelian fields is $\frac{d}{dt}\mathbf{r=} \frac{i}{\hbar}[H,\mathbf{r}]$ and $\frac{d}{dt}\mathbf{s=} \frac{i}{\hbar}\left[ H,\mathbf{s} \right]=\boldsymbol{\Omega}\times\mathbf{s}$, where $\boldsymbol{\Omega}$ is associated with SU(2) gauge potentials [3]. In principle, the amplitude of the ZB effect depends not only on the magnitude of non-Abelian gauge field but also on the initial spin. Also the amplitude shall reach a maximum if the initial spin is perpendicular to $\boldsymbol{\Omega}$.

In optical scenario, our observed ZB effect shall be related to the amplitude of non-Abelian electric field as well as the manner in which the pseudospin is excited. The amplitude of non-Abelian electric field is dependent to the material property. In Figure 3 in the main text, we have observed the change in amplitude as $\theta$ varied, which is a direct evidence of tuning the amplitude through non-Abelian electric field. In Figure S3, we change the initial spin by replacing the excitation source $\mathbf{E}=(0,E_{0},E_{0})$ by $\mathbf{E}=(0,E_{0},{3E}_{0})$, while keeping all other parameters the same with Figure S2(c); the change in amplitude is significant.

Reference

1. Y. Chen, R. Y. Zhang, Z. Xiong, et al., "Non-abelian gauge field optics," *Nat. Commun.,* vol. 10, p. 3125, 2019.
2. F. Liu, T. Xu, S. Wang, Z. H. Hang, and J. Li, "Polarization beam splitting with gauge field metamaterials," *Adv. Opt. Mater.,* vol. 7, p. 1801582, 2019.
3. M. Hasan, C. S. Madasu, K. D. Rathod, et al., "Wave packet dynamics in synthetic non-Abelian gauge fields," *Phys. Rev. Lett.,* vol. 129, p. 130402, 2022.
